# Growth Performance of Broilers as Influenced by Different Levels and Sources of Methionine Plus Cysteine

**DOI:** 10.3390/ani9121056

**Published:** 2019-12-01

**Authors:** Abd Ur Rehman, Muhammad Arif, Muhammad M. Husnain, Mahmoud Alagawany, Mohamed E. Abd El-Hack, Ayman E. Taha, Shaaban S. Elnesr, Mervat A. Abdel-Latif, Sarah I. Othman, Ahmed A. Allam

**Affiliations:** 1 Department of Animal Sciences, College of Agriculture, University of Sargodha, Sargodha 40100, Pakistan; thenutritionist@ymail.com (A.U.R.); arif.inayat@uos.edu.pk (M.A.); globalpeace84@gmail.com (M.M.H.); 2 Department of Poultry, Faculty of Agriculture, Zagazig University, Zagazig 44511, Egypt; dr.mahmoud.alagwany@gmail.com; 3 Department of Animal Husbandry and Animal Wealth Development, Faculty of Veterinary Medicine, Alexandria University, Behira, Rasheed, Edfina 22758, Egypt; ayman.taha@alexu.edu.eg; 4 Department of Poultry Production, Faculty of Agriculture, Fayoum University, Fayoum 63514, Egypt; ss00@fayoum.edu.eg; 5 Department of Nutrition and Veterinary Clinical Nutrition, Faculty of Veterinary Medicine, Damanhour University, Damanhour 22511, Egypt; mervat.abdellatif@vetmed.dmu.edu.eg; 6 Biology Department, College of Science, Princess Nourah Bint Abdulrahman University, Riyadh 11671, BO. Box 24428, Saudi Arabia; sialothman@pnu.edu.sa; 7 Department of Zoology, Faculty of Science, Beni-suef University, Beni-suef 65211, Egypt; allam1081981@yahoo.com

**Keywords:** methionine, levels, sources, growth, carcass, broiler

## Abstract

**Simple Summary:**

The current work evaluated the utilization of different sources of methionine either from DL-methionine (DL-Met) or L-methionine (L-Met) using different concentrations of dietary methionine plus cystine (Met + Cyst) in broiler chickens. Results showed that a better edible meat yield could be obtained by supplementing Met + Cyst at the rate of 80% of the digestible lysine.

**Abstract:**

The objective of this work was to evaluate the utilization of methionine from DL-methionine (DL-Met) and L-methionine (L-Met) with different levels of dietary methionine plus cystine (Met + Cyst) in broilers. The experimental diets were formulated by using three levels of Met + Cyst, i.e., 74%, 77% and 80% of digestible lysine. Met + Cyst was provided either from DL-Met or L-Met. A total of 450 day-old broilers were divided into six groups (five replicates of 15 birds each) in a 3 × 2 factorial arrangement under completely randomized design. Weight gain (WG), feed intake (FI) and feed conversion ratio (FCR) was determined. At the end of the experiment (35 days), two birds from each replicate were slaughtered to determine carcass characteristics and serum homocysteine. Results indicate that the combined effect of L-Met and DL-Met significantly affected (*p* < 0.05) the WG in the starter period and FI in the finisher period. Neither source nor level of methionine influenced (*p* > 0.05) the FI, WG and FCR of broilers during the starter, finisher or overall phase of growth. The interaction between sources and levels of methionine did not influence (*p* > 0.05) the feed intake, weight gain and FCR during the overall phase of growth. Source of methionine had no (*p* > 0.05) effect on carcass characteristics. Methionine levels had a significant effect (*p* < 0.05) on carcass weight, chest weight and thigh weight. The interaction between sources and levels of methionine had a significant (*p* < 0.05) effect on the liver weight. The sources of methionine had significant (*p* < 0.05) effects on the liver and heart weight, while methionine levels significantly influenced (*p* < 0.05) the liver and gizzard weight. Finally, it was concluded that if DL-Met and L-Met are included in feed at a standard level, they are equally effective as a source of methionine for broilers.

## 1. Introduction

Methionine has a vital role in the metabolic functioning of animals and humans, which is why it is also known as functional amino acid. Methionine is considered as the first limiting amino acid in broilers and its deficiency may cause reduced growth performance, metabolic disorder and impaired immune system [1,2]. It plays a vital role in the production of energy through the synthesis of protein; it also enhances the broilers’ livability, efficiency of feed and growth performance [3,4,5]. Also, a methyl group that is provided by sulfur-adenosyl methionine is required for many metabolic reactions such as epinephrine, carnitine, choline and creatine synthesis [5,6]. Synthetic sources of methionine (L-methionine (L-Met), DL-methionine (DL-Met) and DL-2 hydroxy–4-(methyl) butanoic acid (LMA)) are included in commercial broiler feed to optimize the dietary level of methionine. However, synthetic methionine is very expensive and the availability of methionine from different synthetic sources is controversial [7]. The availability of methionine from L-Met, DL-Met and LMA is 100%, 99% and 88%, respectively. L-Met is directly used by the animal as a precursor for protein synthesis and metabolized through the trans-sulfuration pathway to produce cysteine and glutathione [8,9]. Methionine hydroxy analog free acid (MHA-FA) is chemically different from DL-Met because it has a hydroxyl group at the asymmetric carbon atom, whereas DL-Met has an amino group. This chemical difference lowers the bio-availability of MHA compared to DL-Met [10,11].

Different levels of methionine in the diet of poultry have been reported by researchers, ranging from 0.3–1.2% during the initial period and 0.3–0.9% during the growth period of poultry. It has been suggested that commercial poultry production does not need more than 0.38% and 0.50% methionine in grower and starter diets, respectively, for the optimum feed efficiency and growth of broilers, although high rates of methionine are necessary to boost the immune system [12]. Reports regarding the dietary level of methionine are controversial. Kalinowski [13] studied the effect of DL-Met levels (0.32%, 0.38%, 0.44%, and 0.50%) with a constant level of L-cystine (0.40%) on slow and fast growing broilers from 3 to 6 weeks of age, and observed that weight gain was not affected and the feed conversion ratio (FCR) was improved with the highest level of methionine. However, Xie et al. [14] reported that increasing levels of DL-methionine (0.285%, 0.385%, 0.485%, 0.585% or 0.685%) resulted in decreased feed intake and weight gain because of higher plasma homocysteine concentration. This might be related to differences in the source of methionine used. Because of deamination of other amino acids during conversion of D-Met to L-Met, different sources of methionine may perform differently. Ribeiro et al. [8] observed that L-Met addition in broiler diet provided better FCR as compared to DL-Met, and MHA. Lui et al. [15] observed that the bioavailability of MHA-FA was greater than DL-Met. Data regarding the use of different methionine sources with varying dietary Met + Cyst levels are scarce, thus the main objective of this study was to evaluate the utilization of methionine from DL-Met and L-Met with different levels of dietary Met + Cyst in broilers. 

## 2. Materials and Methods

The animal experiment was conducted in accordance with the recommendations and guidelines of the Committee on the Ethics of Animal Experiments of Sargodha University, Sargodha, Pakistan.

### 2.1. Experimental Design, Birds and Diets

The experiment was conducted at the poultry research center at the College of Agriculture, University of Sargodha (Sargodha, Pakistan). A total of 450 day-old broiler chickens (Ross 308-mixed sex) with similar body weight were randomly divided into 6 groups in a 3 × 2 factorial arrangement under completely randomized design (CRD). Each group had five replicates (pens) of 15 birds. Six experimental diets were formulated (Table 1 and Table 2) by using 3 levels of Met + Cyst (74%, 77% and 80% of digestible lysine) and two sources (DL-Met and L-Met) of methionine. Chickens were reared in suitable pens, under the same managerial, hygienic and environmental conditions. Each diet was randomly allotted to each group for five consecutive successive weeks.

### 2.2. Housing and Management

The housing area was cleaned and fumigated before the arrival of the chicks. Fumigation was done by using KMnO_4_ and formalin. Similar management conditions (floor space, temperature, relative humidity, light and ventilation) were provided to all replicates. Feed and water were provided ad libtium.

### 2.3. Growth Performance

Feed intake and weight gain were recorded through the test periods (the starter period corresponds to 1–21 days of age, the finisher period to 22–35 days of age, and the overall period to 1–35 days of age). The feed intake was calculated by the difference between feed supplied and refusal at each period. The feed conversion ratio (FCR) was calculated by dividing feed intake by weight gain [16].

### 2.4. Carcass Evaluation and Serum Homocysteine

At the end of the experiment, two birds of average body weight from each replicate were randomly selected and slaughtered to determine the carcass characteristics (live weight, carcass weight, eviscerated weight, chest weight and thigh weight) and weight of visceral organs (liver, heart and gizzard).

### 2.5. Blood Sampling

Blood samples (*n* = 5) were collected from the wing vein at 35 days of age without anticoagulant for serum separation. Samples were centrifuged at 1435× *g* for 5 min at 4 °C to obtain clear sera, which was collected for homocysteine analysis using chromatographic assay [17].

### 2.6. Statistical Analysis

Data collected were analyzed by using the analysis of variance technique in a 3 × 2 factorial arrangement under CRD. Means of all parameters were separated by using Tukey’s test with the assistance of software (SAS^®^ 9.3 Software).

## 3. Results

### 3.1. Growth Performance

The combined effect of L-Met and DL-Met significantly affected (*p* < 0.05) the weight gain of broilers in the starter period. Neither the source nor levels of methionine influenced (*p* > 0.05) the feed intake, weight gain and FCR of broilers during the starter, finisher or the whole period (Table 3).

Data presented in Table 3 indicate that the interaction between source and levels of methionine had a significant effect (*p* < 0.05) on the feed intake and weight gain during the finisher and starter period, respectively. The results regarding feed intake in the finisher period revealed that the best values (*p* < 0.05) were achieved at an 80% ratio of L-Met. However, no significant differences in feed intake were observed between LM80 and DLM80. During the starter period, the highest values of weight gain (*p* < 0.05) were achieved with a 77% or 80% ratio of L-Met and DLM in comparison with a ratio of 74%.

As shown in Table 4, the interaction between sources and levels of methionine did not influence (*p* > 0.05) the feed intake, weight gain and FCR during the overall phase of growth.

### 3.2. Carcass Characteristics

The source × level of methionine had a significant (*p* < 0.05) effect on thigh weight and non- significant (*p* > 0.05) effect on live weight, carcass weight, after skin removal, eviscerated weight and chest weight (Table 5). The source of methionine had a non-significant effect (*p* > 0.05) on the carcass characteristics of broilers. Level of methionine had a significant (*p* < 0.05) effect on carcass weight, chest weight and thigh weight and a non-significant (*p* > 0.05) effect on live weight, after skin removal and eviscerated weight.

### 3.3. Weight of the Visceral Organs 

As presented in Table 6, the interaction between sources and levels of methionine had a significant (*p* < 0.05) effect on liver weight, while the effect on heart and gizzard weight was non-significant (*p* > 0.05). On the other hand, with regard to liver weight, there was no significant difference between LM80 and DLM80. The sources of methionine had a significant (*p* < 0.05) effect on the liver and heart weight while the effect on the gizzard weight was non-significant (*p* > 0.05), since LM increases liver and heart weight when compared to DLM. Liver and gizzard weights were gradually increased as the levels of methionine increased from 74% to 77% to 80% (*p* < 0.05).

### 3.4. Serum Homocysteine

The combined effect of L-Met and DL-Met had no (*p* > 0.05) effect on serum homocysteine level (Table 6). Neither source nor level of methionine had a significant (*p* > 0.05) influence on serum homocysteine level.

## 4. Discussion

The body weight gain of broilers was significantly increased (*p* < 0.05) in the starter period due to the combined effect of L-Met and DL-Met. These findings of growth performance confirmed the reports of earlier researchers, Ahmed and Abbas [18] who studied the effect of dietary methionine levels above the nutrient requirements of poultry (NRC) [19] recommendation on performance and carcass traits in broiler birds. Four dietary levels of methionine, 0%, 100%, 120% and 130% of the NRC recommendation were used. Weight gain was significantly higher by 110% and 130% of NRC methionine than that of the control diet. Better weight gain with L-Met than DL-Met is also supported by the findings of Katz and Baker [20] who observed that L-Met provided better and more efficient weight gain than D-Met or DL-Met. At a level of supplementation near the requirement, equal efficiency was attained because L-Met is 100% absorbed in the body as compared to DL-Met.

The feed intake and FCR in the starter period remained unchanged by the combined effect of L-Met and DL-Met; this is supported by other researchers [21,22] who have also observed no significant difference in feed intake and FCR due to supplementation of L-Met and DL-Met because when the diet was supplemented with methionine alone, some methionine was converted to cysteine. The presence of small excess amounts of cysteine depressed the feed intake without a proportional reduction in weight gain because the presence of cysteine reduces the metabolic damage. The combined expression of methionine and cystine as sulfur amino acids restricts the efficient use of feedstuff, and also results in inconsistency in requirements. The conversion of methionine to cysteine was nonequivalence [23] and cystine oxidation occurs when it increases beyond the limit, resulting in inefficiency in the accurate estimate of the requirement of individuals. The replacement value for cysteine in broilers that are 3–6 weeks old is 52% [24], however, Wheeler and Latshaw [25] reported 43% and NRC [18] reported 47% as the recommended value. It has been estimated that about 10% of dietary protein is diverted by the broiler in the first 6 weeks to the formation of feathers [26]; this process is high in cysteine [27]. Engler et al. [28] reported that less cysteine is required by male broiler genotypes that are low feathering (L/k^+^) after the age of 3 weeks, and this results in a 15% advantage in the feather weight of the k^+^/k^+^ bird at the age of 48 days [29]. If the nutrients are stored in the feathers then it will not be available for other purposes; while the muscle of breast nourishment rate is reduced by the continuous production of keratin, which limits the supply of nutrients [30]. Therefore, the cystine deficiency results in the reduction in the recovery of breast meat and also decreases the extent of feathering. Our finding of no effects of the sources of methionine on the starter phase of broilers is supported by other researchers [22,31] who observed that L-Met and DL-Met did not affect growth performance due to conversion of DL-Met into L-Met. It seems that lower metabolization of D–amino acid due to the lower amount of D–amino acid oxidase in young broilers may reduce the utilization of higher amounts of DL-Met, which leads to metabolic stress and inhibition of body weight gain in the starter phase. Our findings regarding unaltered feed intake, weight gain and FCR of broilers in the starter period due to different levels of methionine are similar to those of other researchers [32,33], which might be attributed to the satisfaction of methionine requirements at the lower standard level. 

The results regarding feed intake of broilers in the finisher period indicated significant differences (*p* < 0.05) between L-Met and DL-Met. This finding confirmed the reports of earlier researchers [34] who observed better feed efficiency of chicks fed an L-Met diet as compared to DL-Met because the supplementation of either L-Met or DL-Met have beneficial effects on villus development in association with increased glutathione production and levels of total antioxidant capacity, and reduced protein oxidation in the duodenum. Supplementation of L-Met has a better effect on redox status and development of the gut of young chicks as compared with DL-Met.

Our finding of no changes in the weight gain of broilers in the finisher period by the combined effect of L-Met and DL-Met is the similar to other researchers [33,35,36] who observed that L-Met or DL-Met did not significantly influence the weight gain of broilers in the finisher period. This was because when large quantities of methionine are added in the feed, excess methionine is converted into homocysteine and higher amounts of homocysteine in the body reduce the body weight of broilers [37]. No significant differences (*p* > 0.05) were observed by different type of methionine on feed intake, weight gain, and FCR of broilers in the finisher period. This finding confirms the reports of earlier researchers [32,33,38]. Because d-amino acid oxidase, the key enzyme that converts D-Met to L-Met, exists only in the liver and kidney, D-Met is not utilized directly by the cells of the gastrointestinal tract until it is converted to L-Met either in the liver or kidneys. Research has also shown that the expression of this enzyme is very low for young animals. Therefore, L-Met is the only biologically functional form of methionine that is readily utilized by the intestinal cells of young animals. The quantity of methionine had no effect (*p* > 0.05) on the performance of broilers in the finisher period. This finding confirms the reports of earlier researchers [21,22] who observed that levels of methionine had no effect on feed intake, average daily gain, feed efficiency and FCR of broilers because DL-Met is readily converted into the L-isomer by the animal. Also, our finding that L-Met and DL-Met had no combined effect on feed intake, weight gain and FCR during the overall phase of broiler growth is supported by other researchers [33,35,36] who also observed that L-Met or DL-Met did not influence the feed intake, weight gain and overall FCR of broilers. Zhang [7] studied the effect of different dietary methionine source supplementation including L-Met, DL-Met and DL-2-hydroxy-4-(methylthio) butanoic acid (DL-HMTBA) on growth performance. He observed no differences among L-Met, DL-Met and DL-HMTBA for weight gain and feed efficiency. No effect of methionine sources on overall growth of broiler has also been found by other researchers [21,22,31] who observed that L-Met and DL-Met had no effect on the overall phase of broilers.

The results regarding thigh weight indicated that the highest value (*p* < 0.05) was achieved at the 80% ratio of L-Met, while the values achieved at the 80% ratio of DL-Met were lower than L-Met. The significant effect of L-Met and DL-Met on the thigh weight of broilers is supported by other researchers [39,40] who also observed that methionine sources improved the thigh weight of broilers because methionine has a role in the synthesis of creatinine in thigh muscles. No differences (*p* > 0.05) were observed for different types of amino acid on the carcass characteristics of broilers. This finding confirmed the reports of earlier researchers [31,41] who observed that the type of methionine had no effect on live weight, carcass weight, after skin removal, eviscerated weight and chest weight because L-Met is directly absorbed in the body and DL-Met, MHA is first converted into L-Met and then absorbed in the body. Also, Drazbo et al. [42] found that the source of dietary methionine had no effect on carcass yield or breast muscle quality.

The levels of methionine had a significant (*p* < 0.05) effect on carcass weight, chest weight and thigh weight. This finding confirms previous studies [43,44], where levels of methionine had a significant influence on the thigh weight, chest weight and carcass weight. This is because D-Met is oxidatively converted to α-ketoanalogues of L-Met, 2-keto-4(methylthio) butanoic acid (KMB) by the enzyme D-amino acid oxidase, which is a proximal oxidase containing flavin adenine dinucleotide (FAD) as a cofactor. Then, KMB is converted into L-methionine by the transfer of nitrogen from the donor amino acid, which is catalyzed by ubiquitous transaminases. In chickens, many amino acids like glutamic acid, arginine, isoleucine and alanine are used for transamination of KMB [7,11].

Results regarding liver weight indicated that the highest value (*p* < 0.05) was observed at the 80% ratio of L-Met while the values achieved at the 80% ratio of DL-Met were lower than L-Met. The combined effect of L-Met and DL-Met had a significant (*p* < 0.05) effect on the liver weight of broilers. Our finding of unaltered heart and gizzard weight due to the combined effect of L-Met and DL-Met is supported by other researchers [45,46] who observed that DL-Met and herbal methionine had no significant effect on the carcass yield, breast meat and eviscerated weight of broilers. The significant differences observed between the heart and liver weight of birds fed different types of amino acid are corroborated by Ahmed and Abbas [45] who observed that dietary supplementation of methionine significantly affected the liver and heart weight. Ribeiro et al. [35] observed that DL-Met had a significant effect on the gizzard weight in heat stress conditions, which is similar to our findings of differences in gizzard weight due to the type of amino acid.

Unaltered homocysteine due to the combined effect of L-Met and DL-Met in the diet of broilers was supported by the findings of Pillai et al. [47]. They observed that dietary methionine had no effect on hepatic homocysteine remethylation. No effect of the source of methionine on serum homocysteine level of broilers confirms the findings of Harter and Baker [48] who observed that methionine was stored in the plasma of birds fed excess methionine, but plasma levels of homocysteine, cystathionine, and cystine remained unchanged. Haulrik et al. [49] found that high methionine and high protein diet did not significantly increase homocysteine concentration as compared to low methionine and low protein diet, which confirms our findings that there was no change in serum homocysteine level due to different amino acid sources.

On the basis of these results, it may be concluded that if DL-Met and L-Met are included at a standard level in feed, they are equally effective as a source of methionine for broilers. However, better carcass traits may be achieved if Met + Cyst is added at the rate of 80% of digestible lysine.

## Figures and Tables

**Table 1:** Ingredients and nutrients of starter diets (1–21 days).

Ingredients (%)	^1^ Diets					
	DLM74	DLM77	DLM80	LM74	LM77	LM80
Maize	18.85	18.85	18.85	18.85	18.85	18.85
Rice Tips	28	28	28	28	28	28
Soybean Meal	26.23	26.23	26.23	26.23	26.23	26.23
Canola	16	16	16	16	16	16
Rice Polish	4.9	4.9	4.9	4.9	4.9	4.9
Vegetable Oil	2.85	2.85	2.85	2.85	2.83	2.8
Limestone	1.01	1.01	1.01	1.01	1.01	1.01
Di-calcium phosphate	1	1	1	1	1	1
Salt	0.3	0.3	0.3	0.3	0.3	0.3
Lysine	0.408	0.408	0.408	0.408	0.408	0.408
DL-Methionine	0.245	0.282	0.319	0	0	0
L-Methionine	0	0	0	0.245	0.282	0.319
Premix *	0.1	0.1	0.1	0.1	0.1	0.1
L-Threonine	0.085	0.085	0.085	0.085	0.085	0.085
Extra XAP	0.01	0.01	0.01	0.01	0.01	0.01
Extra Phytase	0.01	0.01	0.01	0.01	0.01	0.01
Nutrients (%)						
ME (Kcal/Kg)	2870	2870	2870	2870	2870	2870
Crude Protein	22.1	22.1	22.1	22.1	22.1	22.1
Calcium	1	1	1	1	1	1
Available P	0.45	0.45	0.45	0.45	0.45	0.45
D-Lysine	1.22	1.22	1.22	1.22	1.22	1.22
D-Methionine	0.57	0.606	0.643	0.57	0.606	0.643
Methionine + Cysteine	0.903	0.940	0.976	0.903	0.940	0.976

^1^ LM 74, 77 and 80 and DLM 74, 77 and 80 indicate inclusion of L-methionine and DL-methionine at the rate of 74%, 77% and 80% of digestible lysine, respectively. ME = Metabolizable energy. * Provides per kg of diet: 20 MIU Vitamin A; 5 MIU Vitamin D3; 60 g Vitamin E 50; 2 g Vitamin K3; 6 g Vitamin B2; 45 g Vitamin B3; 12 g Vitamin B5; 5 g Vitamin B6; 12.5 g Vitamin B9; 12.5 g Vitamin B12; 275 g Manganese (MnSO_4_); 150 g Ferrous (FeSO_4_); 200 g Zn (ZnSO_4_); 75 g Cu (CuSO_4_); 75 g Selenium; 4 g Potassium iodide.

**Table 2:** Ingredients and nutrients of finisher diets (22–35 days).

Ingredients (%)	^1^ Diets					
	DLM74	DLM77	DLM80	LM74	LM77	LM80
Maize	22.2	22.2	22.2	22.2	22.1	22
Rice Tips	32	32	32	32	32	32
Soybean Meal	24.33	24.33	24.33	24.33	24.33	24.33
Canola	7.462	7.462	7.462	7.462	7.462	7.462
Rice Polish	4.9	4.9	4.9	4.9	4.9	4.9
Vegetable Oil	3.75	3.75	3.75	3.75	3.75	3.75
Limestone	1.09	1.09	1.09	1.09	1.09	1.09
Di-calcium phosphate	0.8	0.8	0.8	0.8	0.8	0.8
Salt	0.3	0.3	0.3	0.3	0.3	0.3
Lysine	0.458	0.458	0.458	0.458	0.458	0.458
DL-Methionine	0.247	0.282	0.317	0	0	0
L-Methionine	0	0	0	0.247	0.282	0.317
L-Threonine	0.15	0.15	0.15	0.15	0.15	0.15
Premix *	0.1	0.1	0.1	0.1	0.1	0.1
Extra XAP	0.01	0.01	0.01	0.01	0.01	0.01
Extra Phytase	0.01	0.01	0.01	0.01	0.01	0.01
Nutrients (%)						
ME (Kcal/Kg)	3040	3040	3040	3040	3040	3040
Crude Protein	20	20	20	20	20	20
Calcium	0.95	0.95	0.95	0.95	0.95	0.95
Available P	0.44	0.44	0.44	0.44	0.44	0.44
D-Lysine	1.1	1.1	1.1	1.1	1.1	1.1
D-Methionine	0.529	0.563	0.597	0.529	0.563	0.597
Methionine + Cystine	0.814	0.847	0.880	0.814	0.847	0.880

^1^ LM 74, 77 and 80 and DLM 74, 77 and 80 indicate inclusion of L-methionine and DL-methionine at the rate of 74%, 77% and 80% of digestible lysine, respectively. * Provides per kg of diet: 20 MIU Vitamin A; 5 MIU Vitamin D3; 60 g Vitamin E 50; 2 g Vitamin K3; 6 g Vitamin B2; 45 g Vitamin B3; 12 g Vitamin B5; 5 g Vitamin B6; 12.5 g Vitamin B9; 12.5 g Vitamin B12; 275 g Manganese (MnSO_4_); 150 g Ferrous (FeSO_4_); 200 g Zn (ZnSO_4_); 75 g Cu (CuSO_4_); 75 g Selenium; 4 g Potassium Iodide.

**Table 3:** Effect of different sources and levels of methionine plus cystine on growth performance of broilers during the starter and finisher phases.

Treatments	Feed Intake (g)		Weight Gain (g)		FCR (g Feed/g Gain)	
Source × Level	1–21 days	22–35 days	1–21 days	22–35 days	1–21 days	22–35 days
LM 74	1321.5	2105.0 ^b^	934.8 ^b^	1282.4	1.4191	1.9386
LM 77	1342.8	2149.1 ^a,b^	1011.8 ^a^	1209.1	1.3291	1.7873
LM 80	1338.2	2154.9 ^a^	1023.4 ^a^	1282.5	1.3076	1.8108
DLM 74	1335.1	2077.3 ^b^	914.1 ^b^	1129.0	1.3293	1.6395
DLM 77	1320.8	2097.3 ^b^	1005.7 ^a^	1188.9	1.4054	1.8183
DLM 80	1331.5	2150.7 ^a^	1008.1 ^a^	1214.9	1.3225	1.6195
SEM	5.857	15.630	18.002	68.759	0.0262	0.0142
Source						
LM	1334.2	2134.9	990.00	1258.0	1.3524	1.8455
DLM	1329.1	2109.8	984.97	1177.6	1.3519	1.6925
SEM	3.382	9.0243	10.393	39.698	0.0151	0.0601
Level						
74	1328.3	2123.2	970.3	1205.7	1.3742	1.7891
77	1331.8	2129.9	976.4	1199.0	1.3672	1.8028
80	1334.8	2114.0	1015.8	1248.7	1.3150	1.7151
SEM	4.142	11.052	12.72	48.620	0.0185	0.0737
*p*-Values						
Source × Level	NS	*	*	NS	NS	NS
Source	NS	NS	NS	NS	NS	NS
Level	NS	NS	NS	NS	NS	NS

LM 74, 77 and 80 and DLM 74, 77 and 80 indicate inclusion of L-methionine and DL-methionine at the rate of 74%, 77% and 80% of digestible lysine, respectively. ^a,b^ Means sharing different superscripts differ significantly (*p* < 0.05). NS = Non-significant (*p* > 0.05). * = Significant (*p* < 0.05).

**Table 4:** Effect of different sources and levels of methionine plus cystine on growth performance of broilers during overall experimental period (1–35 days).

Treatments	Feed Intake (g)	Weight Gain (g)	FCR (g Feed/g Gain)
Source × Level			
LM 74	3470.5	2288.1	1.6931
LM 77	3447.8	2150.2	1.5739
LM 80	3488.9	2290.6	1.5692
DLM 74	3432.5	2063.8	1.5015
DLM 77	3475.6	2200.7	1.6284
DLM 80	3408.8	2238.3	1.4891
SEM	18.641	78.525	0.0570
Source			
LM	3469.1	2243.0	1.6120
DLM	3439.0	2167.6	1.5397
SEM	10.762	45.337	0.0329
Level			
74	3451.5	2176.0	1.5973
77	3461.7	2175.5	1.6011
80	3448.8	2264.5	1.5292
SEM	13.181	55.526	0.0403
*p*-Values			
Source × Level	NS	NS	NS
Source	NS	NS	NS
Level	NS	NS	NS

LM 74, 77 and 80 and DLM 74, 77 and 80 indicate inclusion of L-methionine and DL-methionine at the rate of 74%, 77% and 80% of digestible lysine, respectively. NS = Non-significant (*p* > 0.05).

**Table 5:** Effect of different sources and levels of methionine plus cystine on carcass characteristics of broilers.

Treatments	Live Weight (g)	Carcass Weight (g)	After Skin Removal (g)	Eviscerated Weight (g)	Chest Weight (g)	Thigh Weight (g)
Source × Level						
LM 74	2051.2	1979.4	1721.0	1329.2	581.60	528.20 ^b^
LM 77	2096.0	2027.2	1801.0	1560.2	642.00	548.00 ^a,b^
LM 80	2368.4	2288.8	2002.8	1747.4	685.00	620.60 ^a^
DLM 74	2067.2	1992.8	1729.0	1804.0	584.60	489.90 ^b^
DLM 77	2157.4	2087.6	1830.4	1491.0	614.60	531.00 ^a,b^
DLM 80	2180.0	2118.6	1792.0	1538.0	643.00	573.80 ^a^
SEM	93.01	85.12	82.45	166.58	27.27	26.68
Source						
LM	2171.9	2098.5	1841.5	1545.6	636.20	552.83
DLM	2134.9	2066.3	1783.8	1611.0	614.0	544.33
SEM	53.70	49.14	47.60	96.17	15.74	15.40
Level						
74	2059.2	1981.1 ^b^	1725.0	1566.6	583.10 ^b^	509.05 ^b^
77	2126.7	2057.4 ^a,b^	1815.7	1525.6	628.30 ^a,b^	539.50 ^a,b^
80	2274.2	2203.7 ^a^	1897.3	1642.7	664.00 ^a^	597.20 ^a^
SEM	65.77	60.19	58.30	117.8	19.28	18.87
*p*-Value						
Source × Level	NS	NS	NS	NS	NS	*
Source	NS	NS	NS	NS	NS	NS
Level	NS	*	NS	NS	*	*

LM 74, 77 and 80 and DLM 74, 77 and 80 indicate inclusion of L-methionine and DL-methionine at the rate of 74%, 77% and 80% of digestible lysine, respectively. ^a,b^ Means sharing different superscripts differ significantly (*p* < 0.05). NS = Non-significant (*p* > 0.05). * = Significant (*p* < 0.05).

**Table 6:** Effect of different sources and levels of methionine plus cystine on visceral organs and serum homocysteine of broilers.

Treatments	Liver (g)	Heart (g)	Gizzard (g)	Serum Homocysteine µmol/L
Source × Level				
LM 74	51.0 ^b^	21.4	65.4	47.40
LM 77	54.1 ^a,b^	21.0	73.0	54.62
LM 80	60.6 ^a^	17.2	78.8	58.74
DLM 74	43.4 ^b^	13.8	69.1	52.46
DLM 77	52.4 ^a,b^	14.0	76.0	44.76
DLM 80	54.0 ^a^	12.6	74.4	52.48
SEM	2.78	1.98	3.67	4.686
Source				
LM	55.523 ^a^	19.867 ^a^	72.400	59.900
DLM	49.933 ^b^	13.467 ^b^	73.167	53.687
SEM	1.6059	1.1470	2.1236	2.706
Level				
74	48.75 ^b^	17.6	67.25 ^b^	50.080
77	52.50 ^a,b^	17.5	74.50 ^a,b^	49.690
80	56.50 ^a^	14.9	76.60 ^a^	55.610
SEM	1.966	1.404	2.600	3.3141
*p*-Value				
Source × Level	*	NS	NS	NS
Source	*	*	NS	NS
Level	*	NS	*	NS

LM 74, 77 and 80 and DLM 74, 77 and 80 indicate inclusion of L-methionine and DL-methionine at the rate of 74%, 77% and 80% of digestible lysine, respectively. ^a,b^ Means sharing different superscripts differ significantly (*p* < 0.05). NS = Non-significant (*p* > 0.05). * = Significant (*p* < 0.05).
